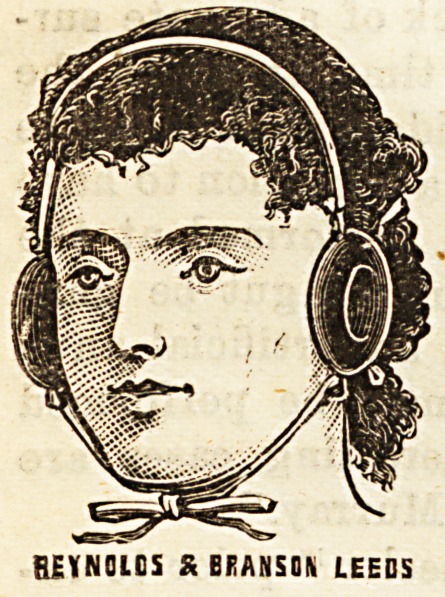# New Appliances and Things Medical

**Published:** 1896-04-04

**Authors:** 


### NEW APPLIANCES AND THINGS MEDICAL.

### PNEUMATIC EAR CUSHION.

Messrs. Reynolds and Branson, surgical instrument makers, 13, Briggate, Leeds, have just brought out a novelty in the shape of "pneumatic ear cushions." These consist of shaped and inflated indiarubber rings, fastened together with tapes across the head and under the chin, intended to be worn in cases of long illness, when the pressure of the ear on the pillow has become painful and is likely to produce sores. The idea is an ingenious one, and the "ear cushions" will be hailed with gratitude by invalids who have experienced pain and discomfort from this cause.

### APENTA WATER.

(Apollinaris Company, London.)

We have received some specimens of this new aperient water from the Apollinaris Company. The water is derived from the Uj Hunyadi springs near Buda Pesth, and from its composition, as indicated by the following analysis of Professor Liebermann, it must be regarded as the finest amongst natural aperient waters known:—

| | |
|---|---|
| Sulphate of soda ... ... ... ... | 15·4320 |
| Sulphate of magnesia... ... ... ... | 24·4968 |
| Sulphate of lime ... ... ... ... | 1·0989 |
| Chloride of sodium ... ... ... ... | 1·8720 |
| Bicarbonate of lime... ... ... ... | 0·8843 |
| Bicarbonate of protoxide of iron ... ... | 0·0189 |
| Silicic acid ... ... ... ... ... ... | 0·0100 |

Other salts are contained in minute traces, but for practical purposes the above analysis may be accepted as complete. It will be noticed that there is a preponderance of sulphate of magnesia, an aperient salt less disagreeable to the palate than the corresponding sodium salt. Apenta water, which should be taken systematically every morning in quantities of about three ounces, should prove a valuable water for those of gouty diathesis, and for those who are afflicted with chronic inflammation or constipation.

### LEMONADE, GINGER BEER, MINERAL WATERS.

(R. Ellis and Sons, Ruthin, North Wales)

We have before had occasion to mention the excellence of the various mineral waters and non-alcoholic beverages prepared by the above firm. The samples which they now forward to us include home-brewed ginger beer in stone bottles, ginger ale (aromatic), seltzer water, ginger ale (dry non-alcoholic), potass water, soda water, lithia and potass water, lithia water, and lemonade. Those who indulge in non-alcoholic drinks seldom have presented to them by one firm so varied a selection from which to choose. Each appears to us to be first-rate of its kind, and, as is not always the case with the manufacturers of such waters, Ellis and Sons can be depended upon to supply water impregnated with the mineral that is indicated on the labels.

### LIQUID PEPTONOIDS, SOLUBLE FOOD, COD LIVER OIL MILK, &c.

(Carnrick and Co. (Limited), 24, Hart Street, Bloomsbury, London.)

The liquid peptonoids which are prepared by the above firm are well known as an invaluable food for invalids whose digestive faculties are impaired. Owing to the predigestive processes to which the constituents have been subjected, the liquid peptonoids can be immediately assimilated by the stomach even in conditions when it cannot exercise for itself the physiological powers of digestion. Bulk for bulk, the peptonoids contain a proportion of nutritive food, which is equalled by hardly any other preparation, but its most valu able feature is that clinically it has proved itself capable o absorption when practically everything else has been re jected by the stomach. The peptonoids are stated to b prepared from concentrated beef, milk, and gluten, a com bination of animal and vegetable proteids, which is wel suited to the physiological needs of the organism.

## Figures and Tables

**Figure f1:**